# Assessment of heavy metal pollution in marine sediments from southwest of Mallorca island, Spain

**DOI:** 10.1007/s11356-022-25014-0

**Published:** 2023-01-05

**Authors:** Pedro Agustín Robledo Ardila, Rebeca Álvarez Alonso, Juan José Durán Valsero, Raquel Morales García, Flor Árcega Cabrera, Elisabeth Lamas Cosío, Sergio Durán Laforet

**Affiliations:** 1 IGME-CSIC, Geological Survey of Spain, Balearic Island Unit, Carrer de Felicià Fuster, 7, 07006 Palma, Spain; 2 IGME-CSIC, Geological Survey of Spain, C/ Ríos Rosas, 23, 28003 Madrid, Spain; 3 Unidad de Química Sisal, Facultad de Química, Universidad Nacional Autónoma de México, Puerto de Abrigo S/N, Sisal, Yucatán 97355 México; 4 Universidad de Málaga, Avda. Cervantes, 2. 29071 Málaga, Spain

**Keywords:** Heavy metals, Marine sediments, Mediterranean Sea, Pollution, Wastewater, Discharge

## Abstract

Anthropogenic activities in urban, agrarian, or industrial areas are the main cause of heavy metals in sediments worldwide. Since the 1960s, there have been submarine discharges through outfalls of poorly treated or untreated wastewater on the south coast of Mallorca island (Mediterranean Sea). In this study, the pollution of marine sediments is analyzed at a great number of points on the south of the seacoast. Heavy metal concentrations of As, Ba, Cd, Cr, Cu, Hg, Ni, Pb, Se, V, and Zn, total inorganic carbon (TIC), sulfur, mineralogy, and grain size were analyzed. The objective is to evaluate the concentrations of toxic substances and their spatial distribution and ranges that can negatively affect marine ecosystems and human health. In addition, the results obtained have been compared with standardized indices for marine sediments, and a regional index has been developed with the background values of heavy metals obtained in the sediments of the study area. To obtain consistent conclusions, concentrations of heavy metals were classified with the Igeo Index. The concentrations of heavy metals obtained show that a great number of samples exceeded the limits considered for uncontaminated sediments according to the index applied. Elements such as Hg, Ba, Pb, and Cu showed high concentrations close to the outfalls and lower concentrations in zones far from these points. To support the assessment, chemical processes such as dissolution or chemical precipitation have been studied. The results also show that marine sediments can be a good trap for chemical elements and a good proxy to analyze the impact of anthropogenic activities in areas heavily pressured by humans, and the risk to the environment and human health.

## Introduction

In recent decades, heavy metal pollution of the marine environment has become a global problem (Burton 2002; Gao et al. 2016; Raj et al. 2008; Romano et al. 2021). Generally, the increase in pollutants is a combination of the absence of clear marine environmental legislation and the increase of pollution from anthropogenic activities such as industry, agriculture, or urbanization. In the Mediterranean Sea, tourism development has become one of the most important activities related to the pollution of marine areas, and heavy metals have negatively affected their natural environment (Elsagh 2019; Romano et al. 2021; Quevedo et al. 2012). In tourist areas like Mallorca island, many pollutants are associated with raw sewage during periods of high population rates, such as the summer months. When treatment plants cannot purify the total volume of wastewater, discharges into the sea incorporate contaminating elements (Elsagh et al. 2021) that interact with sediments and marine ecosystems (Árcega et al. 2014; Belzunce et al. 2004; Cabaço et al. 2008; Reynoldson 1987). This fact causes some harmful substances such as pathogens, heavy metals, oils, and emerging hydrocarbons to return to the marine environment, interacting with the biota and entering the human food chain (Árcega et al. 2021b; Romano et al. 2021; Solaun et al. 2009; Tessier and Campbell 1987; Valdelamar 2018). The marine ecosystem of the southwest of the island of Mallorca is of high ecological value and is formed by a habitat where Possedonia oceanica is the key element of the environmental system. This type of marine plant controls the marine nourishment of the sediment and is vital for the subsistence of other living beings. Calmano et al. (1996) have evaluated the quality of aquatic systems through marine sediments. This confirms that a high level of heavy metals in marine sediments can become a major concern (Árcega et al. 2021a; Choi 2011; Luo et al. 2021; Paneer et al. 2012; Perumal et al. 2021). Several studies have confirmed the high contamination of marine sediments in different regions due to heavy metals (CEDEX 2004; Fatoki and Mathabatha 2001; Sakan 2014).

In Spain, the south coast of the Mallorca island has supported a strong tourist activity since the 1960s. This has required intensive use of freshwater by the resident and foreign population to support activities and infrastructures that produce a significant volume of wastewater. The residual water is discharged along the coastal line through submarine outfalls. In many cases, the discharge of wastewater is done without purification by sewage treatment plants. The most important discharge points in the south of Mallorca are close to the city of Palma. There is evidence of the negative effects of these discharges on the marine ecosystem, such as the retreat of aquatic vegetation, i.e., *Posidonia oceanica* (Borja and Muxika 2005; Gómez-Pujol et al. 2013; IEO 2020), and it could represent a risk to human health (Elsagh et al. 2021). Likewise, previous works have shown the presence of high concentrations in the marine environment of bacterial species, heavy metals, and high values of chemical elements such as nitrate or phosphorus due to the bad quality of wastewater discharges (Cabaço et al. al 2008; Castejón 2011; Islam et al. 2005; Moukhchan et al. 2012; Robledo et al. 2021). The objective of this work is to study the concentrations of heavy metals in marine sediments as well as the concentration of the principal chemical negative substances and their spatial distribution.

### Study area

**Fig. 1:**
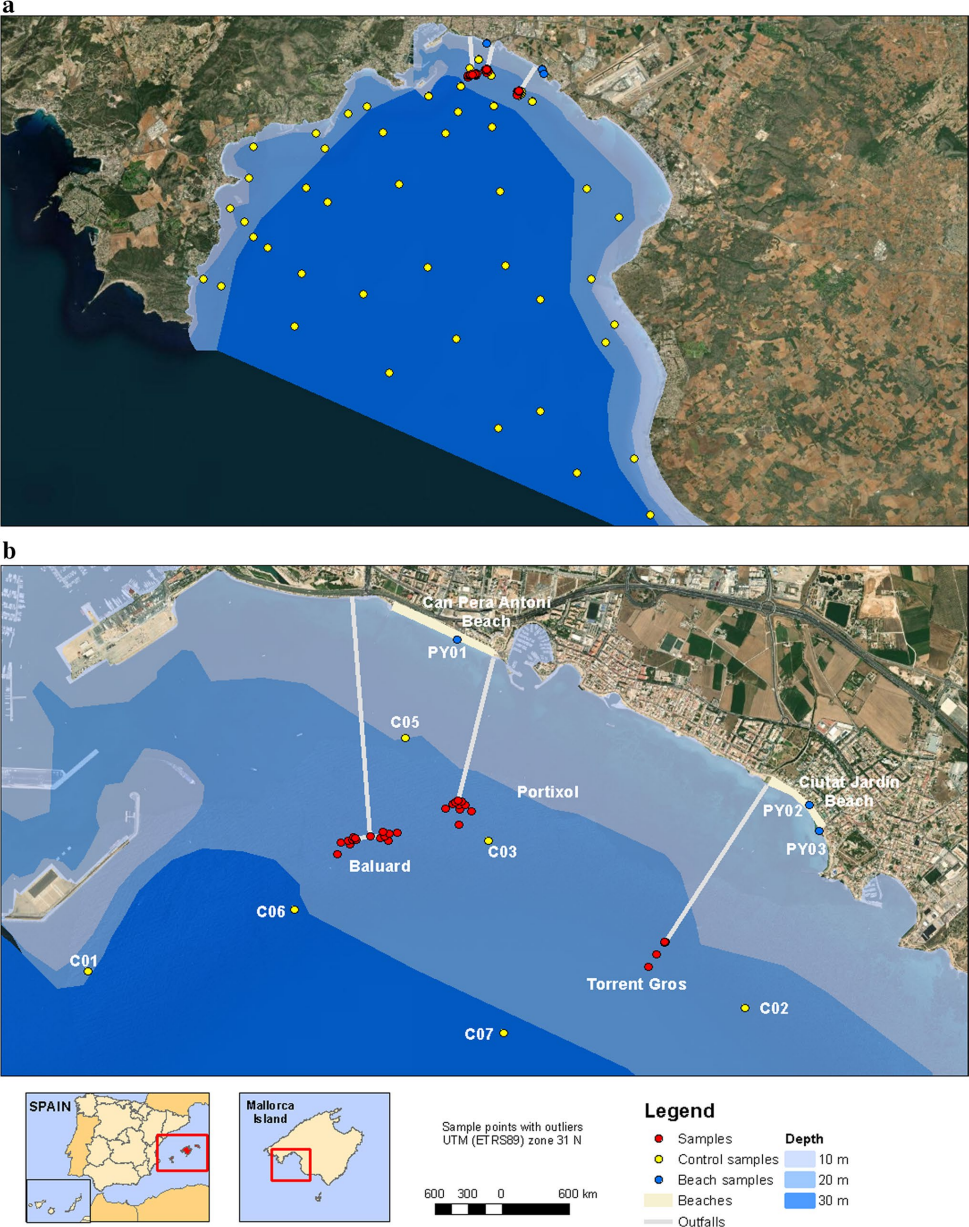
**a** Study area of the marine sediments on the south coast of Mallorca. **b** Zone where the samples with anomalous concentrations are located. In this figure, we show the location of the outfalls and the more important sample stations

The study area is located southwest of the coast of Mallorca (Fig. 1a), between the cape of Cala Figuera to the west and the Xorrigo cliffs to the east. The area is around 160 km^2^, where 450 mm of average annual precipitation is collected. The rivers are only hydraulically active during events of intense rain, spring, and autumn seasons. The most important discharge points in the south of Mallorca are the areas of Baluard to the west of the city of Palma, Portixol in the central part of the coast of Mallorca, and Torrent Gros in the east of the Palma bay (Fig. 1b). These discharge points are located at a depth of 14 m and 1 km offshore. In the study area, the bathymetry shows a regular slope descent of the seabed on the continental margin. The study area ranges from − 10 to − 50 m, and the substrate of the seabed is composed of sands.

## Materials and methods

To assess contamination in marine sediments, the international indices of contamination of marine sediments, grain size, concentration range of chemical elements, mineralogy, and mapping of harmful substances have been used. The results were compared with rates from the US Environmental Protection Agency (EPA) (EPA 1987) and the US National Oceanic and Atmospheric Administration (NOAA) (Buchman 1999). The authors calculate the degree of contamination in marine sediments with global mean background values (Pekey et al. 2004; Savvides et al. 1995; Wedephol 1995). The background is defined as the concentration of heavy metals in sediments undisturbed by human activity. The degree to which current concentrations of heavy metals in sediments exceed background levels is the extent of human contamination (Birch 2016; Pratap et al. 2020; Pekey et al. 2004; Savvides et al. 1995). Nevertheless, by particularities of marine sediments of the coastline in Mallorca, we needed to develop a regional index. This method was developed with the regional background values of the marine sediments of the Mallorca coastline. The result obtained was evaluated according to the geoaccumulation index (Igeo index) (Naifar et al. 2018; Shin and Lam 2001).

In addition, the type of marine sediments on the Majorcan coastline is mainly sand, while gravel and mud sediments are commonly transported by rivers (Font-Muñoz et al. 2017; Servera et al. 2011). The marine substratum of southern Mallorca is composed of bioclastic sands (foraminifera, bivalves, and bryozoans) of variable size (Font-Muñoz et al. 2017), which, in some areas, may have a greater fine fraction or be composed of gravel. Therefore, to determine the mineralogical characteristics, Folk’s ternary diagrams were applied (Folk 1954), which classify the sediments by their granular content of gravel, sand, and mud (silt plus clay). Finally, to have other contamination indicators, chemical parameters such as organic and inorganic carbon (TIC and TOC), phosphorus, and silica were analyzed.

### Sampling

A total of 95 samples of marine sediments were taken in eight field works with the oceanographic vessel using dredger blades and autonomous diving equipment. Some of the samples taken in the area farthest from the wastewater discharge points have been discriminated against, because other factors influence the marine environment, such as greater depth, direction of the currents, or geological substrate. The rest of the samples show high concentrations of heavy metals above the natural values of the sediments. In these places the samples were separated by stations, surrounding the outfalls. At the west coast station, Baluard, 17 samples (B01–B17) were taken, in the central part of the study area, Portixol, 11 samples (B01–B11), and at the eastern station, Torrent Gros, six samples (T01–T06). In addition, six control samples (C01–C06) were between the outfalls and the limits of the study area. Finally, three samples (PY1–PY3) were taken from the beaches to obtain background levels.

### Chemical analyses, grain size, and mineralogy

The chemical parameters have been analyzed using the following techniques:

- Determination of trace elements As, Ba, Cd, Cr, Cu, Ni, Pb, Se, V, and Zn by inductively coupled plasma–mass spectrometry with the Agilent 7500ce equipment based on the EPA 6020 method. Furthermore, the Hg determination is based on EPA 7471 “mercury in solid or semisolid waste.”
- Granulometric analysis followed the UNE 103 100 Standard and is screened in a decreasing direction with the sieves series of UNE, ASTM, and TYLER (IGME 2008).
- Use of atomic absorption spectrophotometry (Beck and Sneddon 2000) to determine the main elements of minerals by X-ray fluorescence.
- TIC was calculated by the difference between the total carbon and the carbon determined after the addition of HCl/FeCl2.

The X-ray laboratory participates twice a year in intercomparison tests. As for the ICP-MS, internal control tests are carried out annually, which consist of repetitions of measurements on the same sample (allows for evaluation of the error made in the treatment of the sample). The analytical precision for the replicate samples achieved was between 0.1 and 2.5%, which was within the acceptable relative standard deviation range that is ≤ 10%.

The granulometry obtained was grouped according to the particle size classes proposed by Wentworth (1922): gravel (> 2 mm), sand (2 mm–63 μm), silt (63–4 μm), and clay (< 4 μm). In addition, the textural classification of the sediment is analyzed using the ternary diagrams proposed by Folk (1954), which allows for the sediment to be classified according to its grain size of gravel, sand, and mud (silt plus clay).

### NOAA, EPA, regional index, and Igeo index

Heavy metal concentrations were evaluated according to reference indices, EPA (EPA 1987), and NOAA (Buchman 1999). EPA limits determine three levels of contamination in marine sediments, and NOAA establishes screening quick reference tables (SQuiRT) related to effects on biological communities (Buchman 1999, 2008).

However, global references are too broad, and a regional index was developed specifically for this study based on local background values of marine sediments. These are the mean values of heavy metal concentrations in beach sand (geochemical signal type, TGS), whose concentrations are not affected by discharges (Table 1). The upper and lower confidence thresholds between the samples from which the TGS is obtained have a barely appreciable variation, ± 0.77 mg/kg. Concentrations above the TGS belong to the threshold called probable pollution effects (PPE). TGS showed very low heavy metal concentrations, in the range of undisturbed by human activities, making them excellent guide levels. Beaches in Mallorca island with low little anthropogenic and tourist pressure have a different geological source of sand nourishment and modify the natural geochemical composition of the sediments. Then a different regional index that is not comparable with the beaches of this study.

**Table 1:** The concentration of heavy metals is calculated in mg/kg (Hg in µg/kg) on the sands of the beaches of Can Pera Antoni and Ciudad Jardin (southwest of Mallorca). These values have been considered background because the concentrations of heavy metals are the natural ranges of the marine sediments in Mallorca

	Ciudad Jardin A	Ciudad Jardin B	Can Pere Antoni	Mean	Standard deviation
As	4.65	4.79	5.51	4.98	0.46
Ba	17.72	14.02	17.33	16.36	2.03
Cd	0.50	0.50	0.50	0.50	0.00
Cr	9.25	7.73	9.34	8.77	0.91
Cu	1.05	0.98	1.47	1.16	0.27
Hg	0.10	0.10	0.10	0.10	0.00
Ni	1.35	1.36	2.36	1.69	0.58
Pb	12.28	9.24	8.79	10.10	1.90
V	7.95	8.39	8.81	8.38	0.43
Zn	8.50	7.95	10.16	8.87	1.15

In addition, the result obtained was evaluated according to the Igeo index (Shin and Lam 2001; Naifar et al. 2018) as a standard evaluation range. This index considers the spatial asymmetry in the concentrations of some heavy metals, with six categories, from the most to the least polluted areas (Table 2). Furthermore, the mineralogical characteristics were studied through Folk ternary diagrams, which classify the sediments by their granular content of gravel, sand, and mud (silt plus clay).

**Table 2:** Range of contamination determined in the Igeo index proposed by Müller (1969)

0	Igeo < 0	Unpolluted
1	0 < Igeo < 1	Unpolluted to moderately polluted
2	1 < Igeo < 2	Moderately polluted
3	2 < Igeo < 3	Moderately to strongly polluted
4	3 < Igeo < 4	Strongly polluted
5	4 < Igeo < 5	Strongly polluted to extremely polluted
6	5 < Igeo	Extremely polluted

### Statistics

Statistical analyzes are based on representing box plots, with the interquartile range, where the median is located and therefore its relationship with the first and third quartiles. The dispersion and symmetry of the heavy metals present in the samples are visually represented through box-whisker diagrams (box or box and whisker plots).

The analysis of the variability of the concentrations of each heavy metal, according to the parametric limits of the EPA, the NOAA, and the regional index has been carried out with a graphic representation that indicates the concentration of each element per sample. The columns located on the left margin of the graph indicate, in different colors (green, orange, or red), their relationship with the thresholds of each index.

### Mapping

To draw the maps, a database has been developed, georeferencing each sample using the ArcGIS 10.8.1 software. The maps have been made using the average nearest neighbor (ANN) relationship, which measures the distance between the centroid of each entity and the location of the centroid of its nearest neighbor (Oncina 1996). This is related to the direction of the dominant ocean currents in the study area. The maps represented have been heavy metals, grain size, TIC, CaCO_3,_ and Si.

## Results

### Chemical analyses of heavy metals

**Fig. 2:**
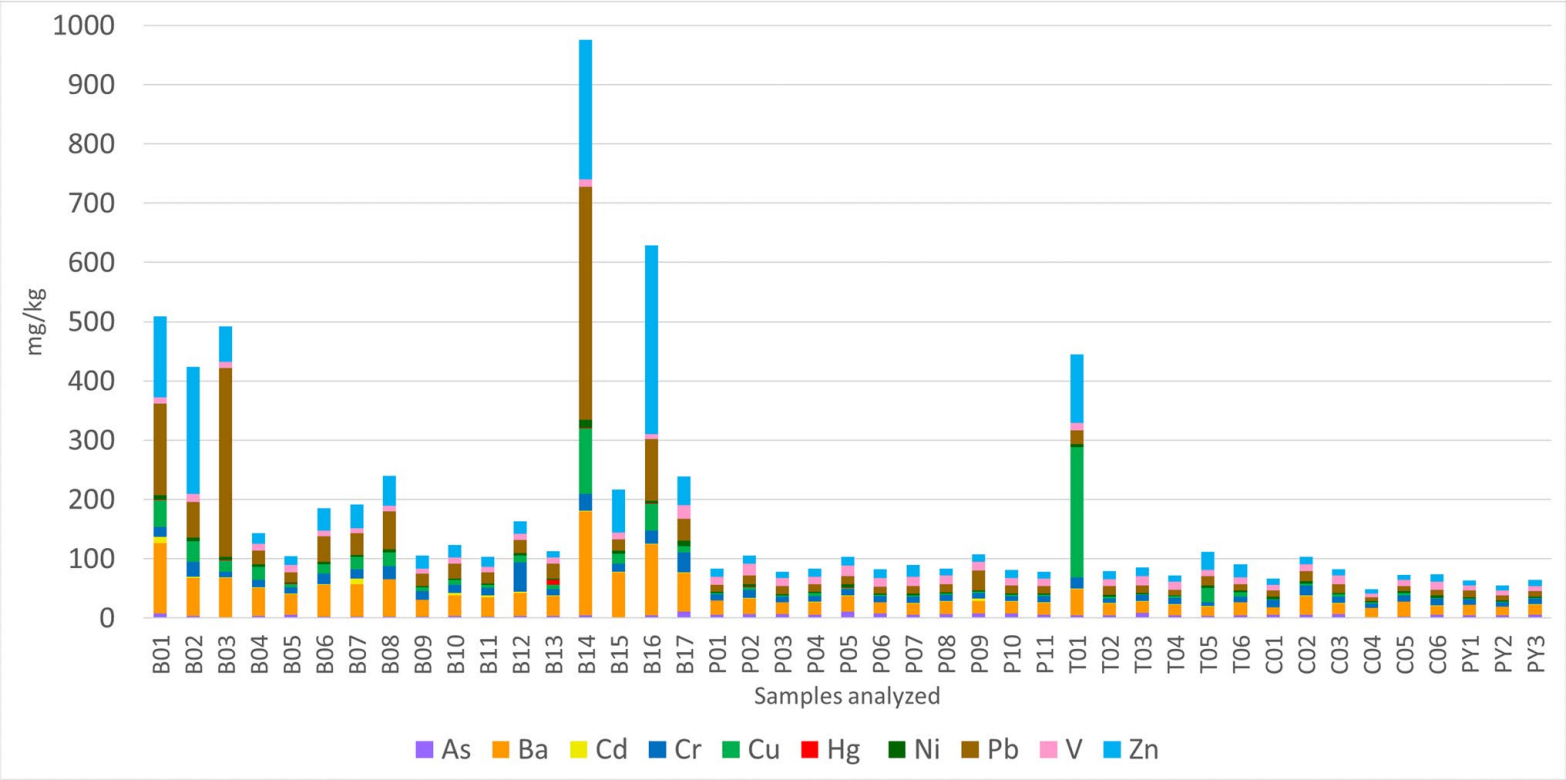
Heavy metal concentration in marine sediment samples from the west (B01–B17), center (P01–P11), and east of the study area (T01–T06); moreover in the graphic the control samples selected (C01–C06) and samples from beaches (PY1–PY3) are represented

Figure 2 shows the results of the heavy metal concentrations. There is great variability in the concentrations between the samples with the highest values in the Baluard station in the west and the lowest values in the Portixol and Torrent Gros stations (Pb, Ba, Zn, and Cu in Baluard are higher in the south of Mallorca). The control samples show moderate concentrations. However, in these samples, some of the heavy metals such as Ba, Cu, Cr, Ni, Pb, or Zn have an anomalous value.

The number of samples that exceed the thresholds defined for each index is shown in Table 3. According to the EPA (1987), highly contaminated values of Cd, Cu, Pb, and Zn have been detected in Baluard and Cu in Torrent Gros, while in Portixol no sample has exceeded the high levels of contamination. As for the NOAA, there were concentrations of heavy metals with probable effects on the biota: Cd, Cu, Hg, Pb, and Zn in Baluard, Cd in Portixol, and Cu in Torrent Gros.

**Table 3:** Number of samples to each threshold according to the parametric limits of the EPA, NOAA, and regional index

		EPA			NOAA				Regional index	
		Not polluted	Moderately polluted	Heavily polluted	Background	TEL	TEL-PEL	PEL	TGS	PPE
B A L U A R D	As				0	15	2	0	14	3
	Ba								0	17
	Cd		15	2	17	9	6	2	7	10
	Cr	14	3	0	5	12	0	0	0	17
	Cu	13	3	1	6	3	7	1	0	17
	Hg				0	6	8	3	1	16
	Ni				15	2	0	0	0	17
	Pb	10	1	6	1	7	6	3	0	17
	V								0	17
	Zn	13	1	3	8	5	3	1	0	17
P O R T I X O L	As				0	7	4	0	0	11
	Ba								0	11
	Cd	0	11	0	0	6	4	1	0	11
	Cr	11	0	0	10	1	0	0	0	11
	Cu	11	0	0	11	0	0	0	0	11
	Hg				0	11	0	0	0	11
	Ni		11	0	0	0	1	10		
	Pb	11	0	0	10	0	1	0	0	11
	V								0	11
	Zn	11	0	0	11	0	0	0	0	11
T. G R O S	As				0	5	1	0	4	2
	Ba								1	5
	Cd	0	6	0	0	6	0	0	0	6
	Cr	6	0	0	5	1	0	0	2	4
	Cu	5	0	1	4	0	1	1	0	6
	Hg				0	2	0	0	6	0
	Ni		6	0	0	0	1	5		
	Pb	6	0	0	5	1	0	0	1	5
	V								0	6
	Zn	5	1	0	5	1	0	0	0	6

About the regional index, at the Baluard and Portixol stations, all the concentrations of heavy metals in the samples are above the parametric limit of the regional standard values. Regarding the Torrent Gros station, the six samples analyzed also exceed the threshold of the regional index, except for Hg.

**Fig. 3:**
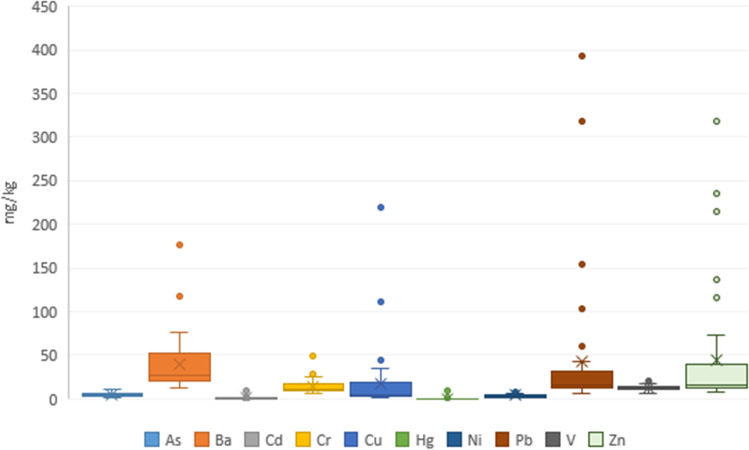
Box plot of each heavy metal analyzed. The 25th and 75th percentiles of the lowest/highest values represent the whiskers, while the 50th percentile shows the interquartile range, 1.5 of the 25/75th percentile. Outliers are shown as circles with the same color as the box. They represent the lower/upper values of the lower/upper whiskers

**Fig. 4:**
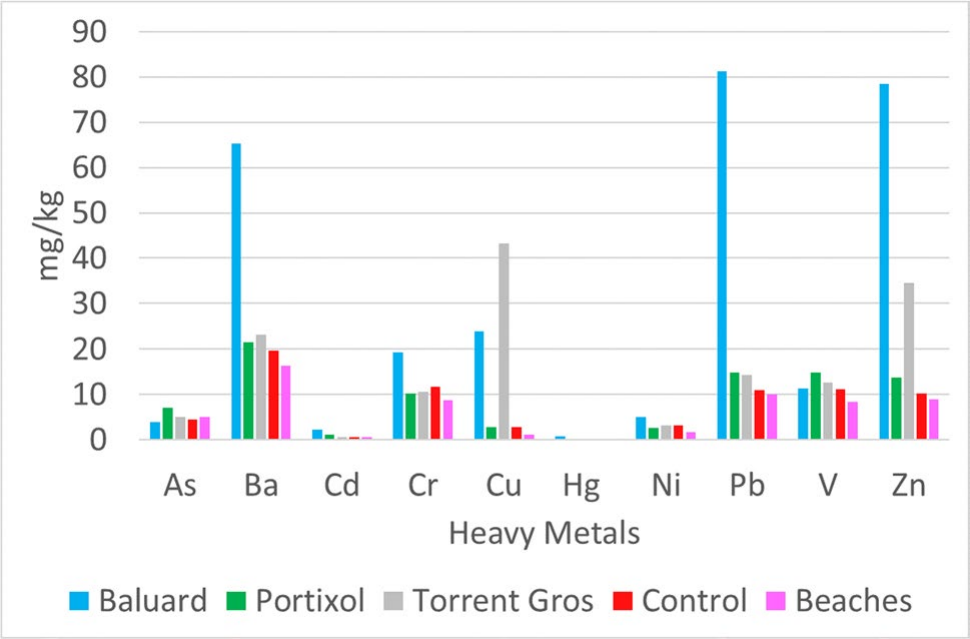
Graph of average heavy metals discretized by sampled areas. There are 5 areas, Baluard to the west, Portixol in the center, Torrent Gros to the east, the control points between the discharge zones, and the beaches where the bottom values are found

The variability of each metal is represented in Figs. 3 and 4. As the results show, there are four metals with high variability: Ba, Cu, Pb, and Zn. In addition, the boxplot of each station indicates that the greatest variability is located in the Baluard station and lowest in the beaches.

**Fig. 5:**
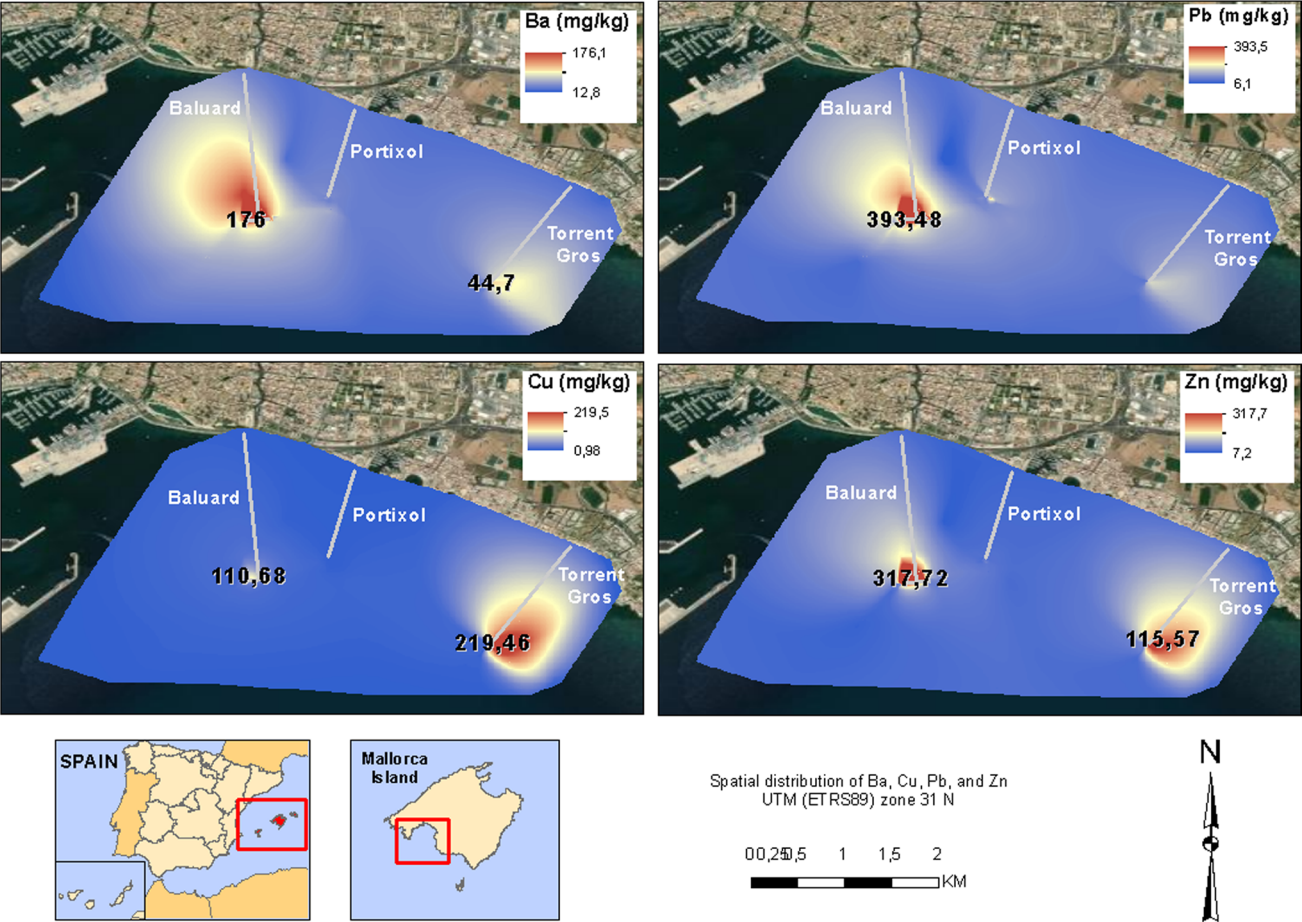
Spatial distribution of Ba, Cu, Pb, and Zn. On the maps, we show the highest values found at the outfall discharge points

The spatial distribution of each heavy metal analyzed is greater at the discharge points and, as shown in Fig. 5, lower far from them. The Baluard station presents the highest concentration values.

### Grain size

**Fig. 6:**
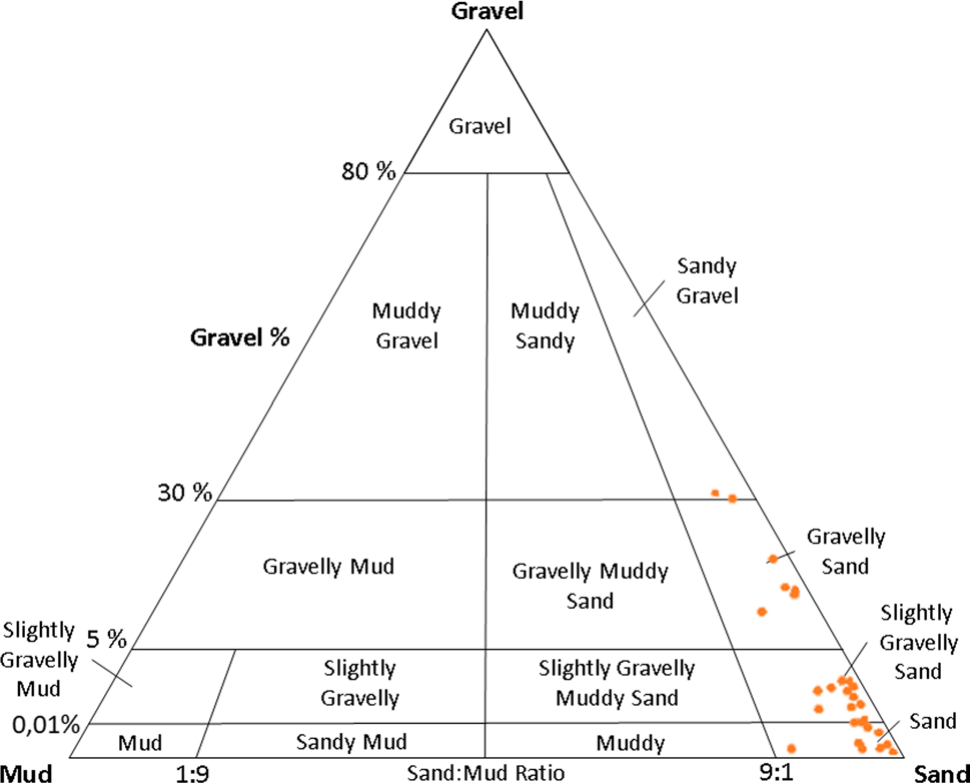
Texture classification of the sediments in the south of Mallorca sea coast from the representation of the frequency percentages of the main granulometric classes in ternary diagrams according to Folk (1954)

The graphical and statistical treatment of the results (Folk 1954) shows that the highest percentage of the total marine sediment is sand (between 0.5 and 2 mm), with a low percentage of gravel fraction (between 2 and 4 mm) and a small amount of mud fraction, which, in this area, includes silt and clay (less than 0.5 mm) (Fig. 6).

**Fig. 7:**
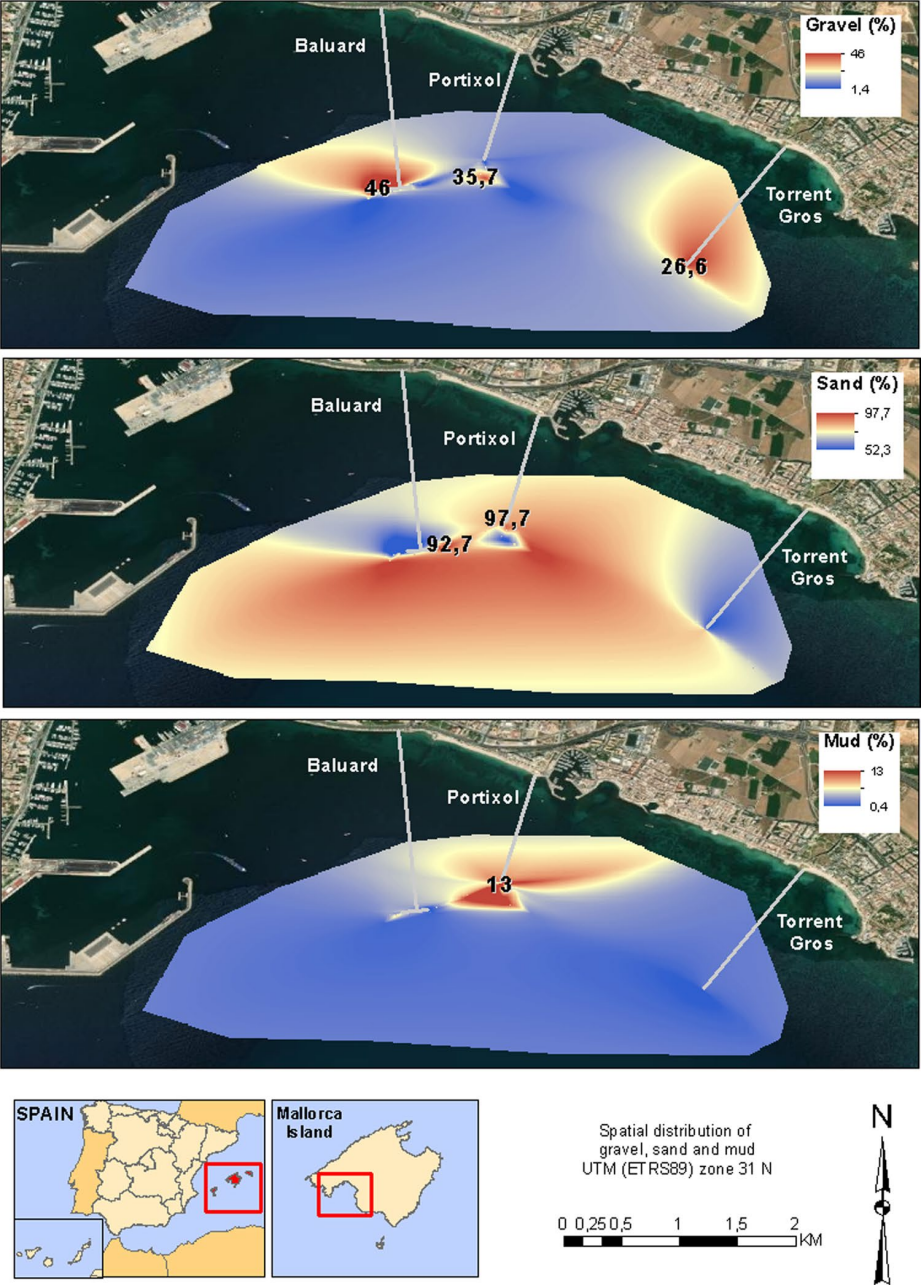
Map of the percentage distribution of **a** gravel, **b** sand, and **c** mud in the south of Mallorca marine coast

This diagram is consistent with the results of the spatial distribution represented in the maps in Fig. 7. The spatial distribution of the sand shows the highest percentages of sand beds that occupy a large part of the study area (between 80 and 90%), especially in the central zone and toward the south. Near the outfalls of Torrent Gros, Baluard, and Portixol, the fraction of sand decreases considerably.

### Mineralogy

The sediment samples were analyzed to show the mineralogical contents of calcite, aragonite, magnesian, calcite, and dolomite. Quartz, halite, gypsum, and feldspars, phyllosilicates, and plagioclases (in micas) have also been detected.

### Calcite, total inorganic carbon, sulfur, and silica

**Fig. 8:**
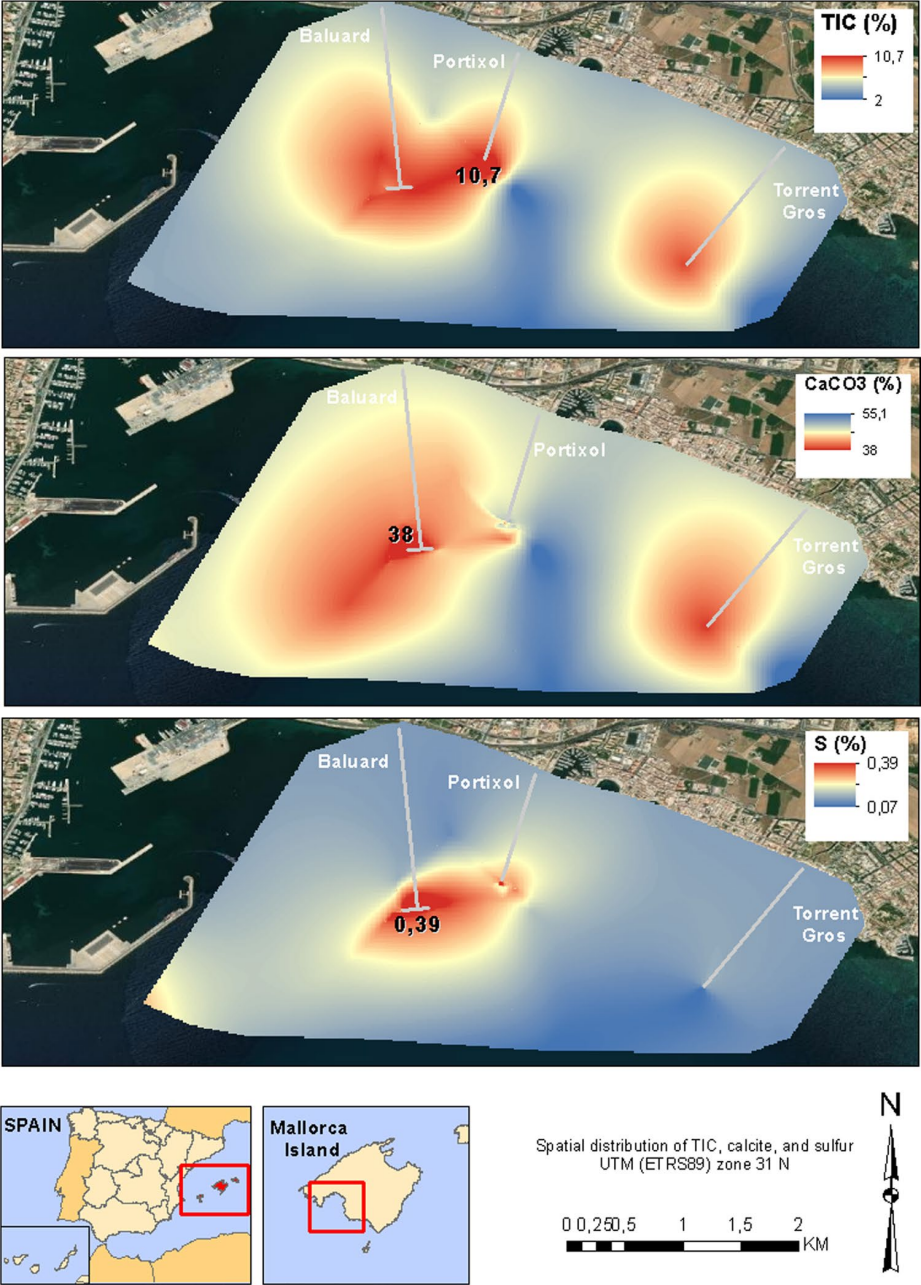
Spatial distribution map of the concentration of TIC, CaCO_3_, and S in the south of Mallorca

The differences in the percentage of CaCO_3_ between the sediment samples from the Baluard, Portixol, and Torrent Gros stations can be observed (Fig. 8). The large number of CaCO_3_ samples from the east and west are less than 50%, lower than the regional value (> 50%). As for the concentrations of S observed in most of the marine sediments in the south of Mallorca, they are around 0.07%. However, in areas close to the Baluard and Torrent Gros outfalls, the values increased strongly, by 0.39 and 0.29%, respectively (Fig. 8). It is similar to the TIC values; although the average concentration in marine sediments does not exceed 2%, it increases at the discharge points until it reaches a value of 9.95 or 10.69%.

## Discussion

### Distribution of heavy metals and contamination levels

**Fig. 9:**
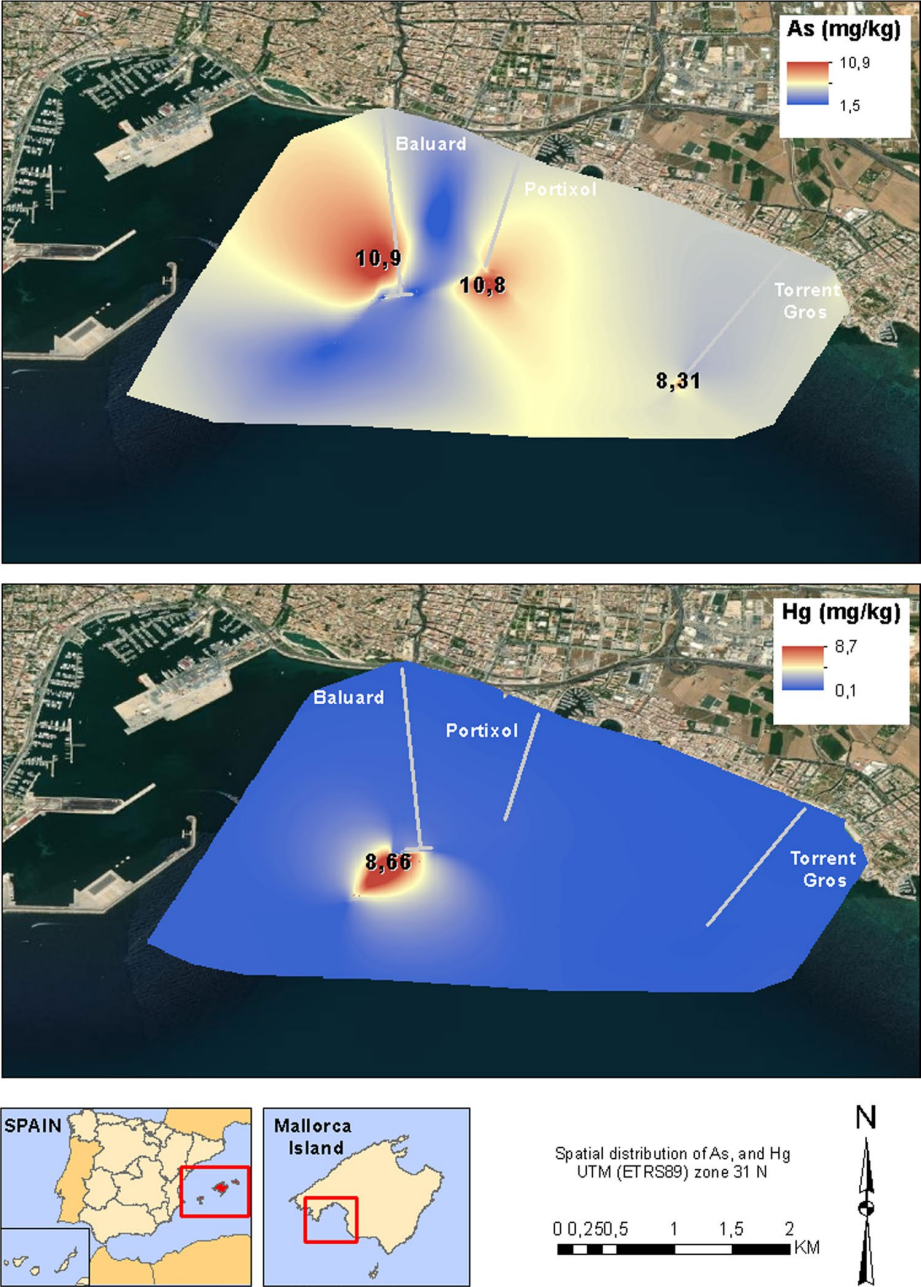
Spatial distribution of As and Hg in the study area

According to the results, there is a significant presence of polluting elements in marine sediments on the south coast of Mallorca island, and these are related to the discharge of untreated wastewater. The concentrations of heavy metals show significant variations between the different areas of the coast. To determine the distribution of contamination by potentially toxic elements, control samples were taken at different points between the outfalls and the limits of the study area. While contamination in the discharge point areas was high for the wide variety of heavy metals analyzed, it decreased in the north of the coast and near the limits of the study area. These samples showed lower concentrations of contaminants, although the values were high compared to the background value of the regional index. Furthermore, the distribution of each heavy metal shows significant variability. The concentrations of As, Ni, and V, although higher near the discharge points of the outfalls, show a homogeneous spatial pattern (Fig. 9). However, the concentrations of Ba, Cd, Cr, Cu, Hg, and Pb are very high in the area surrounding the discharge points.

Related to the levels of contamination, the indices of NOAA and EPA have shown to be a powerful tool to identify high concentrations of pollutants in different areas of the marine coast (Angulo 1996; Buchman 2008). However, the regional index is more accurate, because it allows us to identify polluted areas in relation to the background values (Table 1). Table 1 shows that the differences in TGS values of the marine sediments are < 2 mg/kg, which provides a very good background signal. A great number of samples are found within the PPE limit, which indicates that an extent marine area is contaminated by heavy metals.

**Fig. 10:**
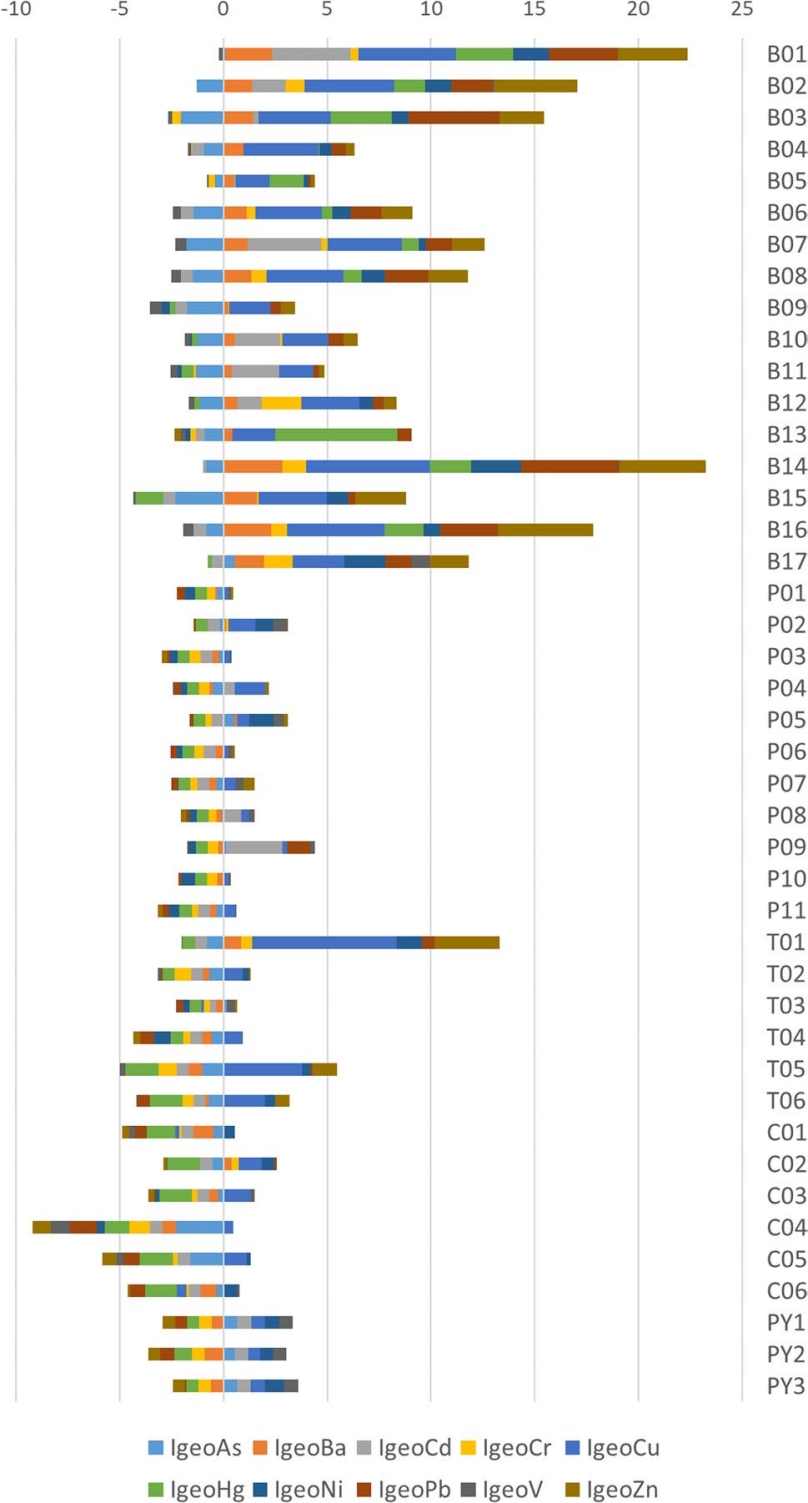
Igeo index results for each sample and each heavy metal considered

There is a wide spatial variability of the concentrations of heavy metals on the southwest coast of Mallorca. According to Shin and Lam (2001), and Naifar et al. (2018), the Igeo index has been applied to compare the degree of contamination with the regional spatial pattern of heavy metals (Fig. 10). Considering the six Igeo categories (Table 2), the results show that the southwest area has the greatest contamination. Heavy metals such as Cu, Cd, Hg, Pb, and Zn showed high levels, reaching extreme values (4–5 and ≥ 5, Igeo index). The most critical contaminants detected in this marine sediment are Hg and Cu, with values higher than 5 (5.85 for Hg and 5.98 and 6.97 for Cu). In addition, the control samples presented anomalous values in Cu, Cr, Ni, Pb, and V (the Igeo index of Cu was greater than 1, and that of Ni was greater than 0.6). This fact may be a good indicator of how local ocean currents distribute heavy metals throughout the marine seacoast (Buccolieri et al. 2006).

### Grain size, mineralogy, and geochemical processes

The anthropogenic impact on coastal marine areas can be observed in the textural and mineralogical variations of sediments (Wang et al. 2020; Valdés et al. 2005). In addition, grain size distribution is closely linked to the concentrations of heavy metals. Sand is the granulometric fraction where the highest concentrations of heavy metals are found. This is based on the hypothesis that heavy metals are incorporated into porosity through chemical precipitation processes. The areas close to the discharge points show an increase in the percentage of gravel, a decrease in the percentage of sand, and a low presence of mud. This fact is linked to three dominant processes: a) the mobilization of the sand fraction by the hydraulic pulse at the discharge points, especially in the southwestern stations; b) the dissolution of the sand fraction due to the aggressive chemistry of the wastewater; c) the transport and deposition of fluvial sediments linked to the Torrent Gros and Na Bárbara rivers (near Portixol) (Fig. 7). The high percentage of mud near the mouth of the rivers is due to the cycles of intense rains when the rivers arrive with a lot of hydraulic energy, transporting a large amount of mud.

The analyzed marine sediments are mainly composed of carbonate rocks, mainly calcite, aragonite, magnesian, calcite, and dolomite (carbonate mineral species). In addition, the presence of quartz has been detected, which is common in this type of sediment due to transport from the different geological units of Mallorca or dragged by the wind and dust from North Africa (Fiola et al. 2005; Robledo 2005). Other minerals typical of these sediments, such as halite, are also part of the main minerals. The gypsum in some of the samples, although not common, could be related to the characteristics of the substrate or geochemical processes that take place in the seawater (sulfur precipitation). On the other hand, the presence of feldspars, phyllosilicates, and plagioclases (micas) is exceptional, and the percentage of this type of mineral in the rocks that constitute the substratum of Mallorca is very low. However, these minerals may be related to the presence of detrital sediments or paleosols, as well as sanidine, muscovite, smectite, biotite, or chlorite, among others (Robledo 2005). In any case, these are deposits made up of mixed minerals, depending on their origin, where carbonated minerals of bioclastic origin predominate.

Wastewater discharges contain elements in dilution and cause complex geochemical processes such as the chemical precipitation of heavy metals (in their ionic form, forming salts) in contact with seawater (Delshab et al. 2017; Andrus 2000; Mashiatullah 2013). Chemical precipitation can be generated by the high diversity of heavy metals in the wastewater, which saturates the dilution, giving rise to chain precipitation according to its solubility product (Robledo et al. 2021; Delshab et al. 2017; Azetsu-Scott et al 2007). According to the TGS limit to quantify contamination in marine sediments, the values of Ba, Cr, Cu, Pb, and Zn have high concentrations. In addition, the concentrations of Hg and As are several orders of magnitude higher than the value of the sediment considered TGS.

Other chemical processes that occur in marine areas influenced by anthropic discharges are the dissolution of CaCO_3_ from marine sediments and the reduction of sulfur (increase in TIC and S) (Delshab et al. 2017). Samples with CaCO_3_ values below 50% indicate that the discharged wastewater is very acidic, dissolving carbonated sediments and increasing the percentage of other poorly soluble minerals such as SiO_2_. In the east zone, the wastewater discharges seem to have a low aggressive chemical and therefore dissolve a little amount of carbonates in the marine sediments (Flores 2011). In this order, we can classify the areas where carbonate dissolution is higher; they have been divided into those with ≥ 50% CaCO_3_ or those with < 50%. The southwest area of the marine coast has been observed with the lowest concentration percentage, 38%. It is observed that in all the discharge points, carbonate values are below 50%, which indicates a significant influence on wastewater discharges and the relationship with sand dissolution processes (mainly carbonate minerals). In addition, SiO_2_ is a very useful mineral to observe the relationship to CaCO_3_. Most SiO_2_ inputs to the marine environment come from continental discharges, both surface and groundwater, and from North African dust storms (Fiola et al. 2005; Sospedraa et al. 2018). CaCO_3_ chemical dissolution processes occur in areas where SiO_2_ increases, close to the wastewater discharge points.

The increase of S and TIC in the areas close to the discharges indicates that an area with low oxygen content and high sulfur reduction capacity has developed in the wastewater discharge areas (Flores 2011; Schulz and Zabel 2006; Booth et al 2012). This fact leads to the precipitation of S, a good tool to detect S-producing bacteriological processes linked to untreated wastewater (Flores 2011). The values of S show high concentrations close to the wastewater discharge points, linked to bacteriological processes of sulfur reduction in an anoxic zone. This also results in the precipitation of sulfur salts that mineralize as octahedral crystal structures (Passier and de Lang 1998; Moran et al. 2019). Anoxic zones are found in the first few meters of water above the seabed. A large number of samples show percentages that exceed 0.2% of S, and the average is 0.19%, high values for marine sediments on the coast of Mallorca. The maximum concentration detected is in the western part, which is 0.39%. The secondary maximum is located in the central area, 0.29%. In the eastern part of the coast, no sample exceeds 0.2%, which may indicate the difference between the types of wastewater discharged between each outfall.

## Conclusions

The anthropic contamination by untreated wastewater discharged on the south coast of Mallorca has been a continuous process shown in the spatial distribution pattern of heavy metals and the dispersion of toxic elements. This pattern shows that the area is more polluted by heavy metals in the marine area close to the beaches, just the most pressured by tourist people.

Wastewater discharges are directly related to the higher concentration of heavy metals in marine sediments. Values are generally high in a great part of the study area, but the highest ones are located right at the discharge points of the outfalls. To the west side of the study area, the concentrations of As, Ba, Cd, Cr, Hg, Ni, Pb, V, and Zn are very high, while to the east of the study area Cu, Cd, Pb, and Zn are the elements with higher concentrations. The granulometry of the sea coast of Majorca is sand. Changes in the amount of sand indicate the existence of geochemical processes that modify the percentages of the grain sizes. Near the outfalls, the amount of sand decreases, and the quantity of gravel and mud increases due to the discharge of raw sewage. However, near the coastline, the increase of gravel and mud is associated with fluvial activity. The more important geochemical process is chain chemical precipitation when wastewater comes into contact with seawater. Due to the saturation of elements in dilution, those are precipitated into the substrate, trapped, and added between the sand and pores. This chemical framework changes the characteristics of the water near the outfalls, taking place significant dissolution processes of CaCO_3_. This is observed in the low quantity of carbonate near the outfalls and the high quantity of SiO_2_.

This research demonstrates that the marine sediment studied on the southwest coast is valuable environmental information. This fact allows for the use of heavy metals in marine sediments as a reliable indicator of anthropogenic impact. Furthermore, the geochemical signature in marine areas highly pressured by anthropic activities tends to deviate extremely from the natural background values. Therefore, there is a high possibility of risk to the environment, the ecosystem, and human health. It is essential to continue and deepen this line of marine research, for example including other touristic areas in the Mediterranean Sea.

## Data Availability

The database used in this study is added as a complementary reference material.
